# Supplementary material for: Development of amplicon deep sequencing markers and data analysis pipeline for genotyping multi-clonal malaria infections
Source: BMC Genomics. 2017 Nov 13;18:864. doi: 10.1186/s12864-017-4260-y (PMC5682641; doi:10.1186/s12864-017-4260-y)
Supplement: Supplementary file 1 — Supporting information. Supplementary text, figures and tables. (DOCX 2111 kb) [file 12864_2017_4260_MOESM1_ESM.docx]

**Additional file 1**

**Protocol: Sequencing library preparation**

Primary PCR

Multiplexed primary PCR was performed in a total volume of 15μl including 2μl template DNA (1:2 diluted), 250nM of each primary primer (GeneWorks Pty Ltd, Australia) and 7.5μl 2xKAPA HiFi HotStart Ready Mix. Cycling conditions were as follows: initial denaturation 95°C for 3 minutes followed by 25 cycles of 20 seconds denaturation at 98°C, 15 seconds annealing at 52°C and 45 seconds elongation at 72°C plus a final elongation of 2 minutes at 72°C.

Nested PCR

Marker-specific nested PCRs were performed in a total volume of 15μl including 1μl primary PCR product diluted 1:10 in dH_2_O, 250nM of the respective nested primer pair (GeneWorks) and 7.5μl 2x KAPA HiFi HotStart Ready Mix (KAPA Biosystems). Cycling conditions were as follows for replicate 1 or 2: initial denaturation 95°C for 3 minutes followed by 15 or 10 cycles of 20 seconds denaturation at 98°C, 15 seconds annealing at 55°C for marker *cpmp* or 56°C for marker *csp* and 45 seconds elongation at 72°C. After 15 or 10 cycles the annealing temperature was increased to 62°C for further 10 or 5 cycles, respectively. Eventually a final elongation of 2 minutes at 72°C was performed. In total, 25 cycles were performed for replicate 1 and 15 cycles for replicate 2.

Pooling of amplicons per sample

Nested PCR products were run on a 1.5% agarose gel for visual inspection of fragment size and quantity. DNA concentration of nested products was estimated in relation to size standard fragments (Solis BioDyne 100bp DNA Ladder). *Cpmp* and *csp* nested PCR products of each sample were pooled in equimolar concentrations. Visual estimation of DNA concentration was difficult as amplicons of marker *csp* and *cpmp* differed in length. To prevent predominance of csp amplicons in the sequencing library due its shorter length, *csp* amplicons were undervalued. This lead to a lower median read coverage for marker *csp* compared to *cpmp*. In case the amplification product was not visible in the agarose gel, equal volumes of both the nested cpmp and csp PCR products nevertheless were pooled.

Sequencing library preparation PCR

PCRs for constructing the sequencing library were carried out in a total volume of 15μl and included 1μl pooled nested products diluted 1:20, 250nM of each sequencing adapter primer and 7.5μl 2xKAPA HiFi HotStart Ready Mix. Cycling conditions were as follows: initial denaturation 95°C for 3 minutes followed by 10 cycles of 20 seconds denaturation at 98°C, 15 seconds annealing at 65°C and 45 seconds elongation at 72°C plus a final elongation of 2 minutes at 72°C.

Pooling of samples

DNA concentrations after these sequencing library PCRs were estimated on a 1.5% agarose gel. All sequencing library PCR products were pooled in equimolar concentrations. This was achieved by pooling equal volumes of all products showing similar band intensity complemented by a pool for PCRs without visible products on agarose gel. These 5 pools were purified with 0.6 volumes of NucleoMag beads (size selection > 300bp) and quantified by Qubit Fluorometer (Thermo Fisher Scientific). Eventually all 5 pools were combined to a final sequencing library by adjusting the volume used from each pool according to its DNA concentration and number of samples combined in a pool.

Sequence library cleanup and sequencing

The expected fragment sizes of the sequencing library were confirmed by Agilent 2200 Tapestation System. The DNA concentration of the final sequencing library pool was quantified by Qubit Fluorometer (Thermo Fisher Scientific). Sequencing was performed on an Illumina MiSeq platform in paired-end mode using MiSeq reagent kit v3 (500-cycles) together with a Enterobacteria phage PhiX Control v3 (Illumina).

**Table S1:** PCR Primer sequence for *msp2* CE genotyping and sequence library preparation.

| **Primer for primary PCR** | |
| --- | --- |
| cpmp_prim_F | CGATACAGGACATATAGA |
| cpmp_prim_R | TTCAATAACATTTACTAGG |
| csp_prim_F | ATCAAGGTAATGGACAAG |
| csp_prim_R | ACTCAAACTAAGATGTGTTC |
| **Primer for nested PCR** | |
| csp_F_Linker | GTGACCTATGAACTCAGGAGTC**AAATGACCCAAACCGAAATGT** |
| csp_R_Linker | CTGAGACTTGCACATCGCAGC**GGAACAAGAAGGATAATACCA** |
| cpmp_F_Linker | GTGACCTATGAACTCAGGAGTC**CATAAGTCATTAAAATTTATGGAT** |
| cpmp_R_Linker | CTGAGACTTGCACATCGCAGC**CGTTACTATCAAGATCGTTAATATC** |
| **Primer for msp2 CE genotyping** | |
| msp2_S2_fw | GAAGGTAATTAAAACATTGTC |
| msp2_S3_rev | GAGGGATGTTGCTGCTCCACAG |
| msp2_S1-fw | GCTTATAATATGAGTATAAGGAGAA |
| msp2_FC27-rev | GCATTGCCAGAACTTGAA |
| msp2_3D7-rev | CTGAAGAGGTACTGGTAGA |
| **Primer for sequence library PCR (XXXXXX=barcode)** | |
| Forward | AATGATACGGCGACCACCGAGATCTACACTCTTTCCCTACACGACGCTCTTCCGATCTXXXXXXXXGTGACCTATGAACTCAGGAGTC |
| Reverse | CAAGCAGAAGACGGCATACGAGATCGGTCTCGGCATTCCTGCTGAACCGCTCTTCCGATCTXXXXXXXXCTGAGACTTGCACATCGCAGC |

| **Forward barcode** | | **Reverse barcode** | |
| --- | --- | --- | --- |
| Fwd_1 | TAGATCGC | Rev_1 | TAAGGCGA |
| Fwd_2 | CTCTCTAT | Rev_2 | CGTACTAG |
| Fwd_3 | TATCCTCT | Rev_3 | AGGCAGAA |
| Fwd_4 | AGAGTAGA | Rev_4 | TCCTGAGC |
| Fwd_5 | GTAAGGAG | Rev_5 | GGACTCCT |
| Fwd_6 | ACTGCATA | Rev_6 | TAGGCATG |
| Fwd_7 | AAGGAGTA | Rev_7 | CTCTCTAC |
| Fwd_8 | CTAAGCCT | Rev_8 | CAGAGAGG |
| Fwd_13 | TGGTGGTA | Rev_9 | GCTACGCT |
| Fwd_14 | TTCACGCA | Rev_10 | CGAGGCTG |
| Fwd_15 | AGCACCTC | Rev_11 | AAGAGGCA |
| Fwd_16 | CAAGGAGC | Rev_12 | GTAGAGGA |
| Fwd_17 | ATTGGCTC | Rev_13 | ATGCCTAA |
| Fwd_18 | CACCTTAC | Rev_14 | ACGCTCGA |
| Fwd_19 | CTAAGGTC | Rev_15 | AGTCACTA |
| Fwd_20 | GAACAGGC | Rev_16 | ATCCTGTA |
|  |  | Rev_17 | CGCATACA |
|  |  | Rev_18 | CTGGCATA |
|  |  | Rev_19 | GATAGACA |
|  |  | Rev_20 | GCTAACGA |
|  |  | Rev_21 | GTGTTCTA |
|  |  | Rev_22 | TCCGTCTA |
|  |  | Rev_23 | CCTAATCC |
|  |  | Rev_24 | GACAGTGC |

**Table S2:** Summary of mismatch rates for linker sequences, marker primers used in primary and nested amplification and for amplicons^1^ of markers *cpmp* and *csp* generated from controlled mixtures of two *P. falciparum* strains 3D7 and HB3

|  | **Linkers**  **%** | **Primers** | | **Amplicons** | |
| --- | --- | --- | --- | --- | --- |
|  |  | **cpmp**  **%** | **csp %** | **cpmp**  **%** | **csp**  **%** |
| **Min** | 0.00 | 0.00 | 0.00 | 0.00 | 0.00 |
| **1^st^ Qu.** | 0.00 | 0.00 | 0.03 | 0.06 | 0.07 |
| **Median** | 0.08 | 0.09 | 0.21 | 0.15 | 0.18 |
| **Mean** | 0.12 | 0.28 | 0.71 | 0.38 | 0.46 |
| **3^rd^ Qu.** | 0.19 | 0.20 | 0.42 | 0.35 | 0.43 |
| **Max** | 1.93 | 10.92 | 22.01 | 15.76 | 18.13 |

^1^ Mismatch rate was calculated relative to 3D7 and HB3 reference sequence.

**Table S3:** Percent of sequence reads clustering to 3D7 reference sequence, percent of PCR artefacts and percent of singleton reads in serial dilution of culture strain 3D7.

| **Parasitaemia**  **per μl** | ***cpmp*** | | | | ***csp*** | | | |
| --- | --- | --- | --- | --- | --- | --- | --- | --- |
|  | **3D7^1^**  **%** | **PCR artefact**  **%** | **Singletons**  **%** | **Coverage** | **3D7^1^**  **%** | **PCR artefact**  **%** | **Singletons**  **%** | **Coverage** |
| 50,000 | 93.3 | 0.3 | 6.4 | 6,758 | 86.4 | 5.2 | 8.4 | 1,623 |
| 5,000 | 92.2 | 0.2 | 7.6 | 2,382 | 85.3 | 4.9 | 9.8 | 1,374 |
| 500 | 91.9 | 0.2 | 7.9 | 3,751 | 85.4 | 5.0 | 9.6 | 3,725 |
| 50 | 93.3 | 0.0 | 6.7 | 165 | 83.0 | 6.1 | 10.9 | 540 |
| 5 | 66.7 | 0.0 | 33.3 | 6 | 87.1 | 0.0 | 12.9 | 70 |

^1^ Percent of reads that cluster with 3D7 reference sequence.

**Table S4**: **Detectability of the minority clone in defined ratios of *P. falciparum* strains HB3 and 3D7 for marker *cpmp*.** Read counts clustering correctly with 3D7 and HB3 haplotypes, as well as read failed to cluster with 3D7 and HB3 haplotypes: false haplotype CPMP-15 (below cut-off criteria), singleton and obvious PCR artefacts (indels and chimeras).

| **Ratios in mixtures** | **3D7** | | **HB3** | | **Singleton** | | **Indels** | | **Chimera** | | **CPMP-15** | | **Coverage** |
| --- | --- | --- | --- | --- | --- | --- | --- | --- | --- | --- | --- | --- | --- |
| **HB3:3D7** | **n** | **%** | **n** | **%** | **n** | **%** | **n** | **%** | **n** | **%** | **n** | **%** | **n** |
| 1:1 | 14,103 | 34.6 | 23,399 | 57.4 | 3,067 | 7.52 | 107 | 0.26 | 92 | 0.23 | 0 | 0.00 | 40,768 |
| 1:10 | 9,854 | 75.6 | 2,141 | 16.4 | 987 | 7.57 | 39 | 0.30 | 15 | 0.12 | 1 | 0.01 | 13,037 |
| 1:50 | 4,400 | 88.8 | 156 | 3.15 | 394 | 7.95 | 3 | 0.06 | 0 | 0.00 | 0 | 0.00 | 4,953 |
| 1:100 | 12,093 | 90.9 | 204 | 1.53 | 966 | 7.26 | 48 | 0.36 | 0 | 0.00 | 0 | 0.00 | 13,311 |
| 1:500 | 5,130 | 90.8 | 27 | 0.48 | 476 | 8.43 | 16 | 0.28 | 0 | 0.00 | 0 | 0.00 | 5,649 |
| 1:1000 | 2,780 | 91.5 | 7 | 0.23 | 251 | 8.26 | 1 | 0.03 | 0 | 0.00 | 0 | 0.00 | 3,039 |
| 1:1500 | 51,680 | 92.5 | 60 | 0.11 | 3,876 | 6.94 | 268 | 0.48 | 3 | 0.01 | 0 | 0.00 | 55,887 |
| 1:3000 | 6,863 | 92.5 | 7 | 0.09 | 518 | 6.98 | 29 | 0.39 | 0 | 0.00 | 0 | 0.00 | 7,417 |

**Table S5**: **Detectability of the minority clone in defined ratios of *P. falciparum* strains HB3 and 3D7 for marker *csp*.** Read counts clustering correctly with 3D7 and HB3 haplotypes, as well as read failed to cluster with 3D7 and HB3 haplotypes: false haplotype CSP-9 (below cut-off criteria), singleton and obvious PCR artefacts (indels and chimeras).

| **Ratios in mixtures** | **3D7** | | **HB3** | | **Singleton** | | **Indels** | | **Chimera** | | **CSP-9** | | **Coverage** |
| --- | --- | --- | --- | --- | --- | --- | --- | --- | --- | --- | --- | --- | --- |
| **HB3:3D7** | **n** | **%** | **n** | **%** | **n** | **%** | **n** | **%** | **n** | **%** | **n** | **%** | **n** |
| 1:1 | 3,126 | 34.7 | 4,550 | 50.5 | 812 | 9.01 | 497 | 5.52 | 24 | 0.26 | 0 | 0.00 | 9,009 |
| 1:10 | 2,544 | 76.1 | 339 | 10.2 | 270 | 8.08 | 186 | 5.57 | 2 | 0.06 | 0 | 0.00 | 3,341 |
| 1:50 | 12,170 | 82.7 | 424 | 2.88 | 1,201 | 8.16 | 890 | 6.05 | 26 | 0.18 | 0 | 0.00 | 14,711 |
| 1:100 | 9,996 | 83.5 | 269 | 2.25 | 1,063 | 8.88 | 644 | 5.38 | 3 | 0.03 | 0 | 0.00 | 11,975 |
| 1:500 | 2,948 | 84.0 | 16 | 0.46 | 377 | 10.75 | 166 | 4.73 | 1 | 0.03 | 0 | 0.00 | 3,508 |
| 1:1000 | 1,548 | 85.7 | 4 | 0.22 | 163 | 9.02 | 92 | 5.09 | 0 | 0.00 | 0 | 0.00 | 1,807 |
| 1:1500 | 20,381 | 86.3 | 19 | 0.08 | 1,866 | 7.90 | 1,349 | 5.71 | 1 | 0.00 | 3 | 0.01 | 23,619 |
| 1:3000 | 1,970 | 85.0 | 1 | 0.04 | 211 | 9.10 | 136 | 5.87 | 0 | 0.00 | 0 | 0.00 | 2,318 |

**Table S6:** Multiplicity of infection of 37 field sample measured by length polymorphic marker *msp2* and SNP polymorphic markers *cpmp* and *csp*.

| **MOI** | ***msp2***  **n** | ***cpmp***  ***n*** | ***csp***  ***n*** |
| --- | --- | --- | --- |
| 1 | 12 | 10 | 19 |
| 2 | 14 | 14 | 16 |
| 3 | 5 | 6 | 2 |
| 4 | 4 | 2 |  |
| 5 | 2 | 5 |  |
| Mean | 2.2 | 2.5 | 1.5 |

**Figure S1:** **Genomic distribution of single nucleotide polymorphism in sequenced alleles of *P. falciparum* gene PF3D7_0104100 (*cpmp* marker)**. The top panel represents alleles of global origin (MalariaGEN *P. falciparum* Community Project, 2016). The bottom panel shows expected heterozygosity values for sliding windows of 100bp across the entire gene. Red box highlights region selected for amplification.

**Figure S2: Genomic distribution of single nucleotide polymorphism in sequenced alleles of the *P. falciparum* circumsporozoite protein *(csp)*.** The top panel represents alleles of global origin (MalariaGEN *P. falciparum* Community Project, 2016). The bottom panel shows expected heterozygosity values for sliding windows of 100bp across the entire gene. Red box highlights region selected for amplification.


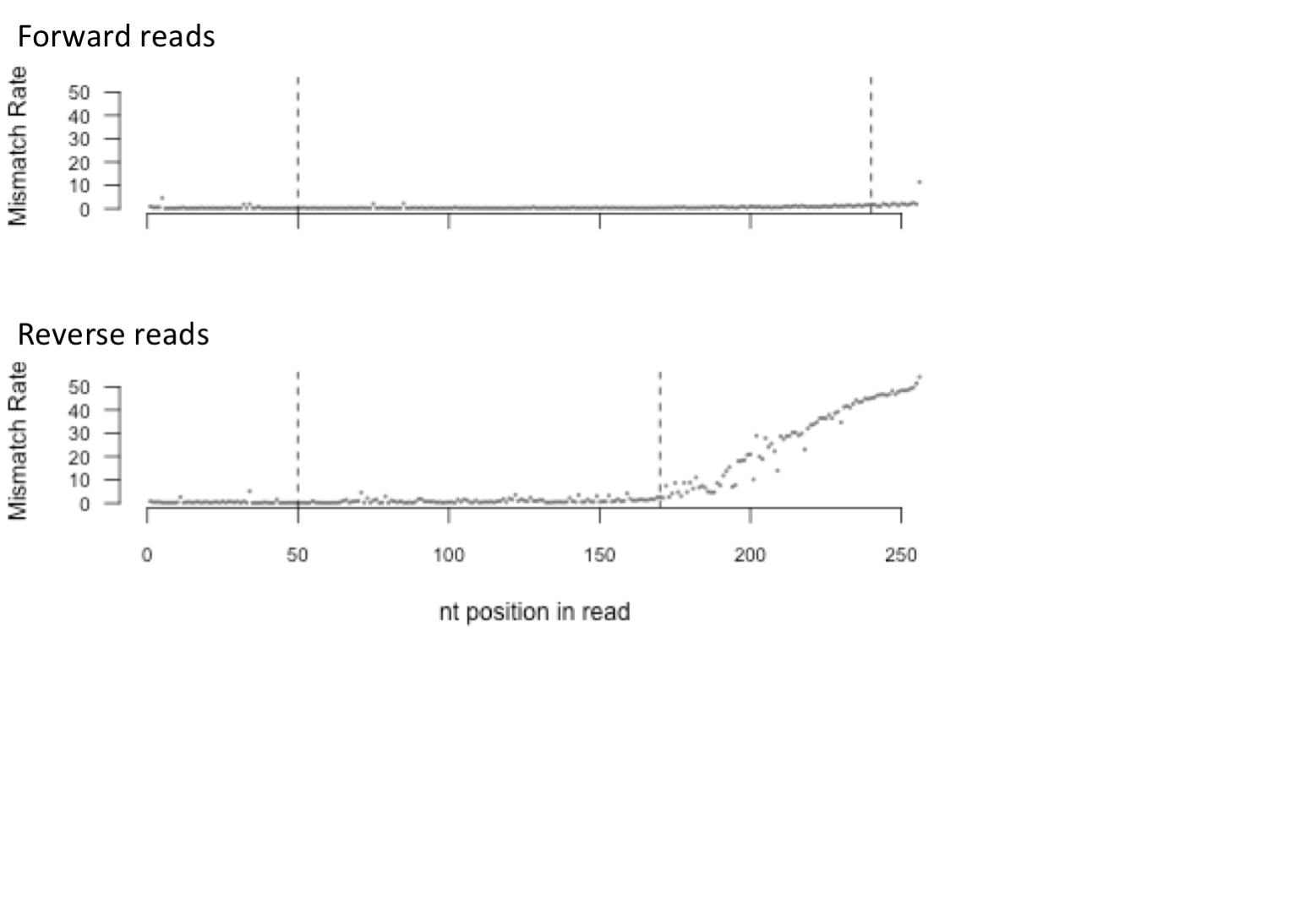


**Figure S3**: **Average mismatch rate per nucleotide position in reads of spiked in control DNA *phiX*.** X-axis: nucleotide position in *phiX* read. Y-axis: mismatch rate with respect to *phiX* reference sequence. Each data point represents the average mismatch rate of all *phiX* reads at a given nucleotide position.


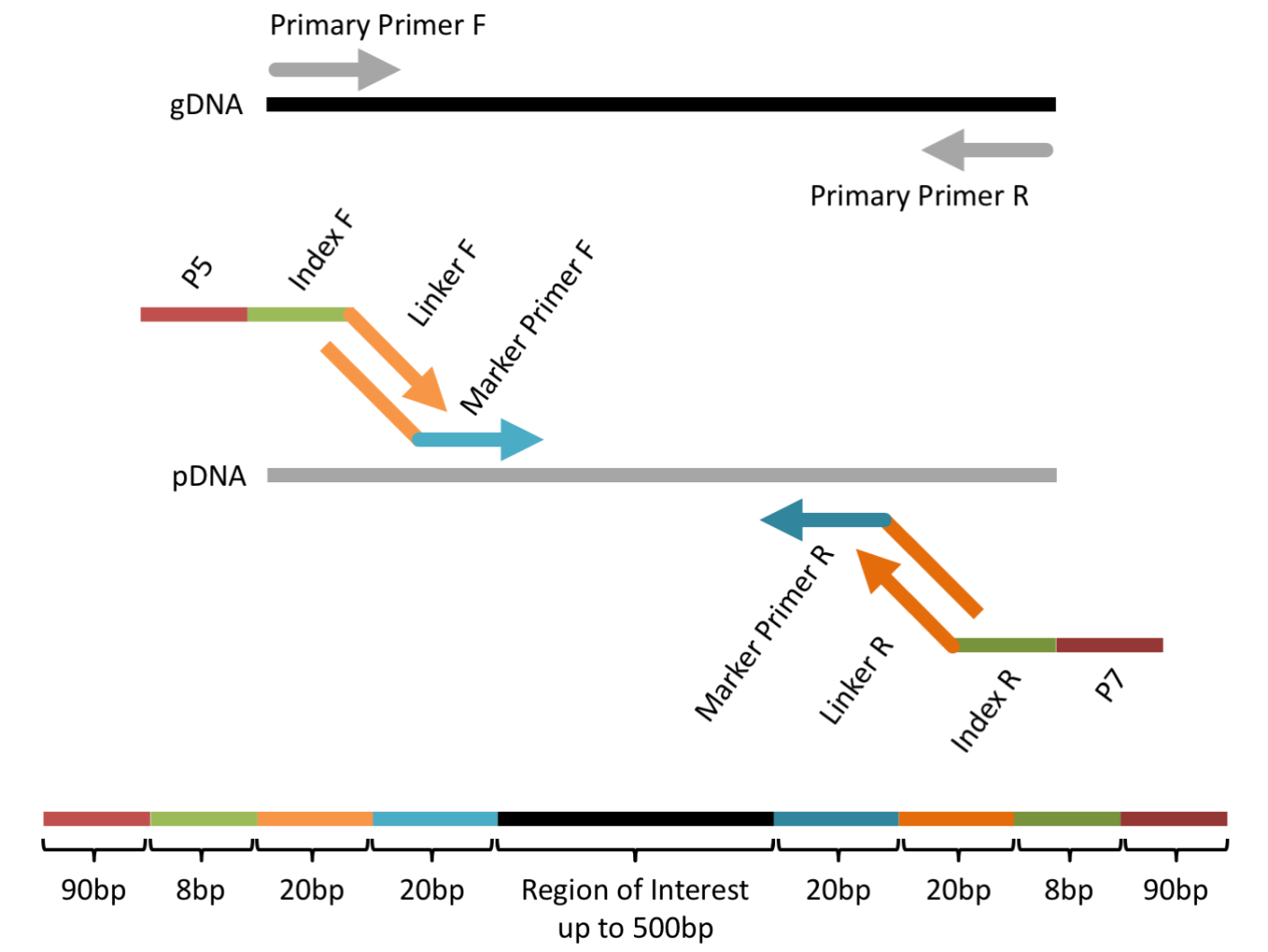


**Figure S4:** **Design of the amplicon sequencing library**. Primary primers target the gene of interest. Primary PCR is followed by nested PCR using marker-specific primers that carry F and R linker sequences at their 5’ ends. The primers for the final round of amplification target the F and R linker sequences. These primers carry sample-specific indices (barcodes) plus Illumina sequencing adapter P5 and P7 at their 5’ ends. The line at the bottom indicates the sizes of the various elements.


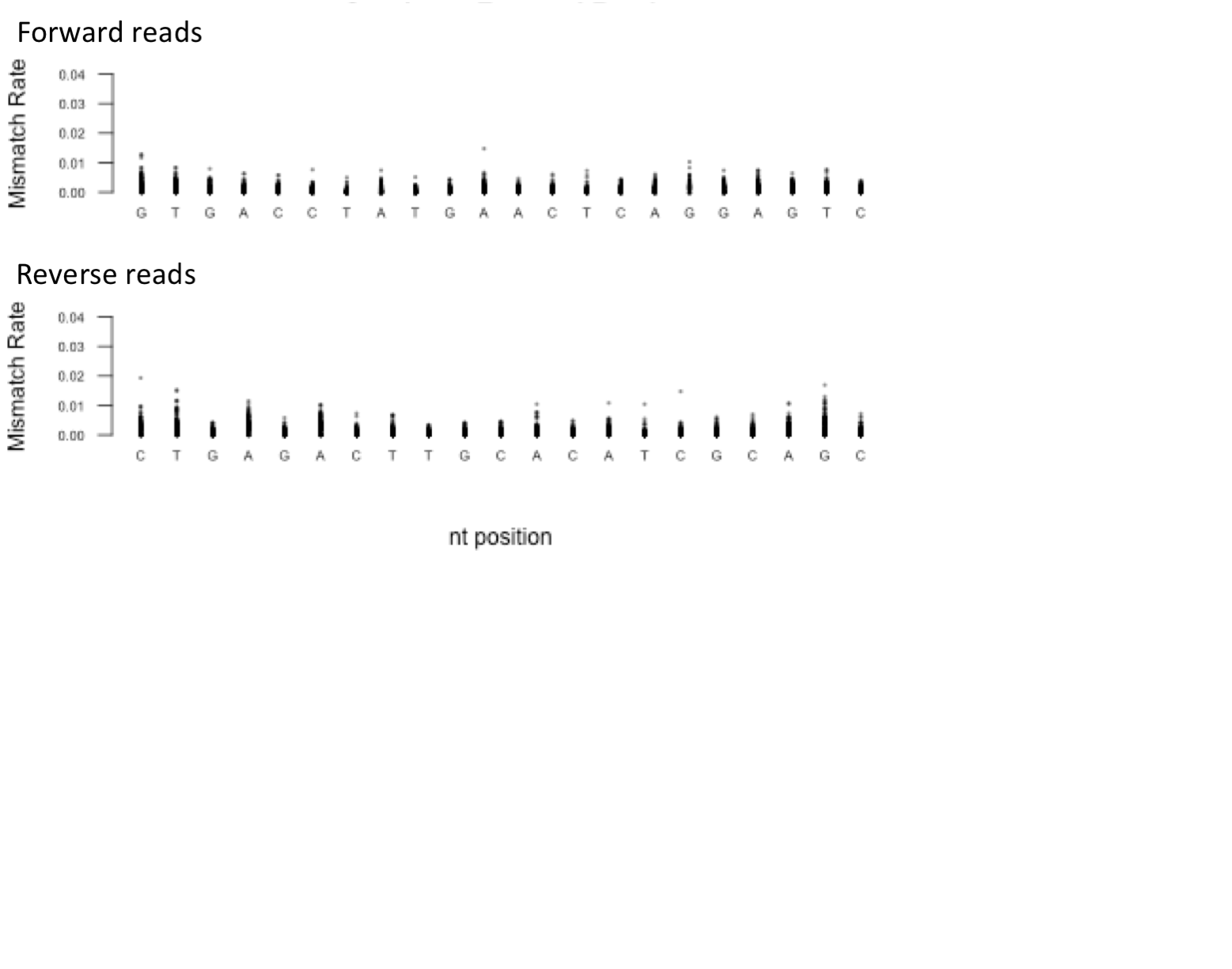


**Figure S5:** **Observed mismatch rate at each nucleotide position in forward and reverse reads of linker sequence.** Data derived from all samples analysed. Each data point represents the mean mismatch rate of all reads from an individual sample. X-axis: nucleotides of forward and reverse linker (5’ to 3’). Y-axis: mismatch rate with respect to known linker sequence.

**Figure S6: Mismatch rate per nucleotide position in forward and reverse primers of markers cpmp and csp**. Data derived from all samples analysed. Each data point represents the mean observed mismatch rate of all reads from an individual sample. Red data points: control samples (P. falciparum culture strains); black data points: field samples; X-axis: nucleotides of forward and reverse primers (5’ to 3’); Y-axis: mismatch rate with respect to the known primer sequences.

**
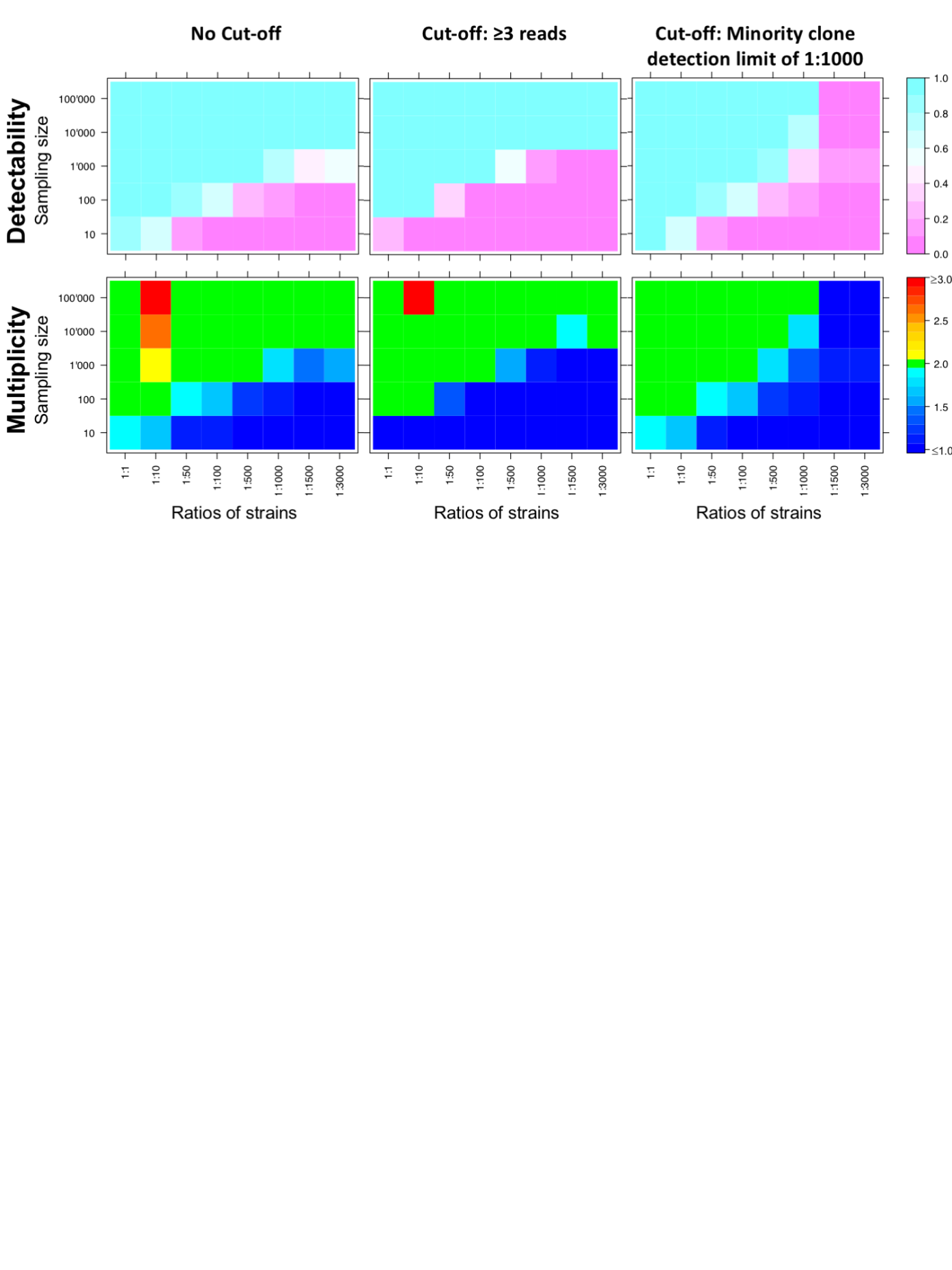
**

**Figure S7: Simulation of the detectability of a minority clone (top panel) and of measured multiplicity of infection (bottom panel) by bootstrapping for marker *cpmp.*** Cut-off settings: no cut-off (left panel); ≥3 read per haplotype (middle panel); minority clone detection limit of 1:1000 (right panel). Samples were drawn from reads of defined mixtures of *P. falciparum* strains 3D7 and HB3. X-axis represents ratios of strains 3D7 and HB3. Y-axis indicates the sampling size (number of draws from the sequence reads (coverage >3000) for each mixture of strains. Sampling was repeated 1000 times to estimate the mean detectability of a minority clone.

**
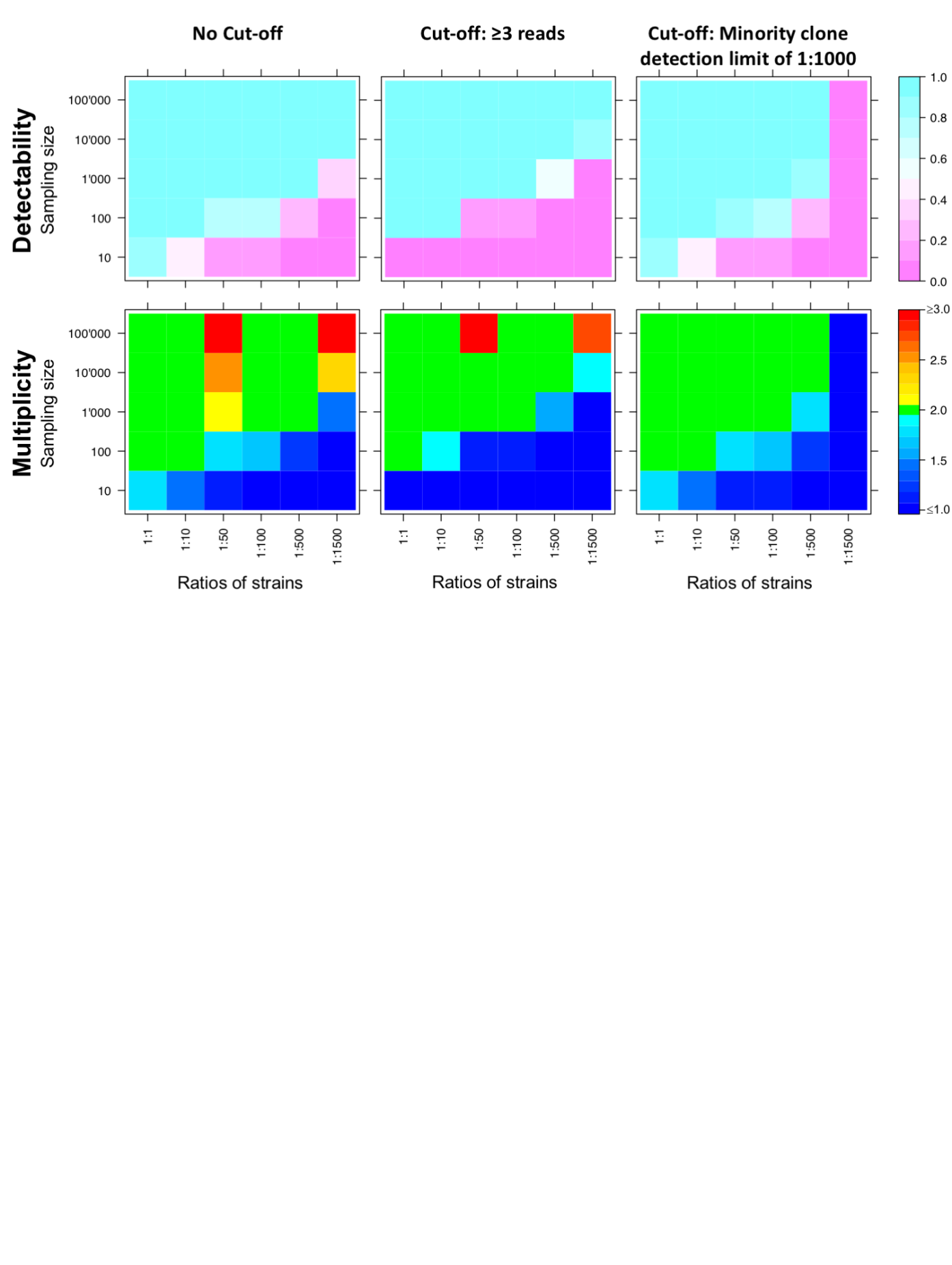
**

**Figure S8: Simulation of the detectability of a minority clone (top panel) and of measured multiplicity of infection (bottom panel) by bootstrapping for marker *csp.*** Cut-off settings: no cut-off (left panel); ≥3 read per haplotype (middle panel); 0.1% minority clone detection limit of 1:1000 (right panel). Samples were drawn from reads of defined mixtures of *P. falciparum* strains 3D7 and HB3. X-axis represents dilution ratios of strains 3D7 and HB3. Y-axis indicates the sampling size (number of draws from the sequence reads (coverage >3000) for each mixture of strains. Sampling was repeated 1000 times to estimate the mean detectability of a minority clone.

**Figure S9: Comparison of genotyping by length-polymorphic marker *msp2* and amplicon sequencing of markers *cpmp* and *csp* exemplified in 1 field sample.** Capillary electropherograms (CE) and dendrograms represent the raw data of markers *msp2*-CE, *cpmp* and *csp* (two top panels). Quantification of haplotypes and final multiplicity call (two bottom panels). Grey shading indicates haplotypes and reads filtered out by cut-off settings (example discussed in detail in results section, paragraph “Validation of amplicon sequencing in field samples”).

**Figure S10:** **Reproducibility of amplicon sequencing in field samples.** Haplotype calls that passed default cut-off criteria were compared between replicates to investigate reproducibility. In grey: number of haplotypes detected in both replicates, in red: number of haplotypes detected only in a single replicate. Inserts present frequency distributions below 1% at a higher resolution.
